# Haemodialysing babies weighing <8 kg with the Newcastle infant dialysis and ultrafiltration system (Nidus): comparison with peritoneal and conventional haemodialysis

**DOI:** 10.1007/s00467-014-2923-3

**Published:** 2014-08-15

**Authors:** Malcolm G. Coulthard, Jean Crosier, Clive Griffiths, Jon Smith, Michael Drinnan, Mike Whitaker, Robert Beckwith, John N. S. Matthews, Paul Flecknell, Heather J. Lambert

**Affiliations:** 1 Department of Paediatric Nephrology, Great North Children’s Hospital, Newcastle, NE1 4LP UK; 2 Clinical Measurement and Engineering Unit, Department of Medical Physics, Freeman Hospital, Newcastle, NE7 7DN UK; 3 Department of Paediatric Anaesthesia, Freeman Hospital, Newcastle, NE7 7DN UK; 4 School of Mathematics and Statistics, Newcastle University, Newcastle, NE1 7RU UK; 5 Comparative Biology Centre, Medical School, University of Newcastle upon Tyne, Newcastle, NE2 4HH UK; 6 South Park House, South Park, Hexham, NE46 1BS UK

**Keywords:** Infant, Acute renal failure, Chronic renal failure, Renal replacement therapy

## Abstract

**Background:**

To compare the efficacy of the Newcastle infant dialysis and ultrafiltration system (Nidus) with peritoneal dialysis (PD) and conventional haemodialysis (HD) in infants weighing <8 kg.

**Methods:**

We compared the urea, creatinine and phosphate clearances, the ultrafiltration precision, and the safety of the Nidus machine with PD in 7 piglets weighing 1–8 kg, in a planned randomised cross-over trial in babies, and in babies for whom no other therapy existed, some of whom later graduated to conventional HD.

**Results:**

Two babies entered the randomised trial; 1 recovered rapidly on PD, the other remained on the Nidus as PD failed. Additionally, 9 babies were treated on the Nidus on humanitarian grounds: 3 because of failed PD, and 3 with permanent kidney failure later converted to conventional HD. We haemodialysed 10 babies weighing between 1.8 and 5.9 kg for 2,475 h during 354 Nidus sessions without any clinically important incidents, and without detectable haemolysis. Single-lumen vascular access was used with no blood priming of circuits. The urea, creatinine and phosphate clearances using the Nidus were around 1.5 to 2.0 ml/min in piglets and babies, and were consistently higher than PD clearances, which ranged from about 0.2 to 0.8 ml/min (*p* ≤ 0.0002 for each chemical). Ultrafiltration was achieved to microlitre precision by the Nidus, but varied widely with PD. Fluid removal using conventional HD was imprecise and resulted in some hypovolaemic episodes requiring correction.

**Conclusion:**

The Nidus can provide HD in the Pediatric Intensive Care Unit (PICU) and outpatient intermittent HD without blood priming for babies weighing <8 kg, It generates higher dialysis clearances than PD, and delivers more precise ultrafiltration control than either PD or conventional HD.

## Introduction

Dialysing small babies is challenging for many reasons. Vascular access for haemodialysis (HD) modalities is problematic as the size of the central venous line (CVL) required for adequate blood flow is disproportionately large for the size of the baby especially when a double lumen line is needed (Poiseuille’s law: flow is proportional to the fourth power of the internal radius).

Peritoneal dialysis (PD) is frequently used to support infants after open-heart surgery [1–3], and sometimes to treat very-low-birthweight babies [4, 5] where conventional HD is typically insurmountably challenging [6]. Larger critically ill infants with multi-organ failure are often treated with a variety of continuously delivered HD modalities (continuous renal replacement therapy, CRRT) [7]. Most babies with chronic renal failure are treated using PD [8, 9], with HD only used for bridging [10].

Peritoneal dialysis is technically simpler than HD [6, 10, 11]; there is no lower size limit for its use, but complications are common in the smallest patients [4–6, 11]. Ultrafiltration (UF) is unpredictable [10], and chemical clearance (including ammonia) less efficient [7, 12], especially in unstable babies who develop splanchnic vasoconstriction and who also risk developing necrotising enterocolitis. This renders PD impossible, as do abdominal surgery and congenital abdominal wall defects.

Conventional HD and CRRT machines cannot control fluid balance better than ±30 ml/h [13], and therefore are not licensed for babies weighing <8 kg (or approved for use in children of <20 kg in the USA). The recommended minimum 7-Fr, dual-lumen vascular access lines and continuous 40 ml/min blood flows [7] are difficult to achieve in the smallest babies. Their relatively large circuit volume (≥60 ml) produces sudden dilution of blood on commencing treatment if primed with crystalloid, and increases the risk of anaemia with circuit loss. Blood priming risks exposing the baby abruptly to aberrant chemical and pH changes, which are reduced by pre-dialysing the circuit [6, 14]. Even in infants, exposure to blood transfusions may increase the risk of developing tissue-type sensitisation, which may affect transplant matching later [15]. The CARPEDIEM device has only just become available with a miniaturised conventional circuit (27 ml) for babies as small as 2.5 kg [16]. Its fluid pump imprecision is ±7.5 %.

In 1995, we therefore designed a novel HD circuit, which operated by different principles. It was driven by syringes, and uncoupled the baby’s blood flow capacity from the requirements of the dialysis filter [17]. In 2005, we reported the results of automating this as a miniaturised machine (circuit volume 13 ml), with which we treated four babies weighing between 800 g and 3.4 kg, using a single-lumen access line, and without the need for blood-priming [18]. We have subsequently developed this device into the Newcastle infant dialysis and ultrafiltration system (Nidus). Here, we compare the efficacy of the Nidus with that of PD in piglets and in babies, and consider its safety and clinical value.

## Materials and methods

### Subjects

#### Piglets

We simultaneously measured the chemical clearances and UF rates produced by PD and the Nidus in anaesthetised piglets weighing between 1 and 8 kg, in a non-recovery study in accordance with the Animals (Scientific Procedures) Act, 1986, whilst infusing urea and creatinine to simulate renal failure. We studied one animal to evaluate unforeseen problems before repeating it in 6 further piglets to minimise the numbers of animals used. To test UF control, we used PD dialysate glucose concentrations of 1.36 to 3.86 %, and set the Nidus to operate at UF rates between 0 and 40 ml/h. We measured serial plasma haemoglobin concentrations to detect haemolysis.

#### Babies

We measured dialysis clearances up to twice daily in infants weighing ≤8 kg who were being treated by the Nidus and/or PD by collecting effluent dialysate fluid when bloods were being tested for clinical reasons. We measured the plasma haemoglobin daily for the first two days to detect haemolysis.

We studied two groups of babies. The RCT group were infants who would normally have received PD, typically after open-heart surgery, but who instead were to be treated sequentially with both PD and the Nidus on alternate days for 4 days, in a cross-over controlled trial where the starting modality was randomised after obtaining informed parental consent. Vascular access was obtained through the 20-gauge lumen of a routinely sited 4.5-Fr triple-lumen central line. The study had National Research Ethics Service (NRES) approval (11/YH/0449) and was conducted according to the 1964 Declaration of Helsinki. The COMP group consisted of babies who were treated on compassionate grounds because their clinicians judged that their best chance of survival was to be dialysed on the Nidus. This had approval from our hospital’s Clinical Governance and Quality Committee, and families were fully informed about its experimental nature before they signed consent for its use. Assessments were also carried out in any of the COMP infants who were subsequently managed with PD.

#### Adult volunteer

In order to re-engineer the Nidus for some babies’ requirements, such as increasing the sampling speed, we needed to test it for safety before clinical use. We undertook this on an adult volunteer using an antecubital vein, with NRES approval and fully informed consent.

### Dialysis methods

#### The Nidus

**Fig. 1:**
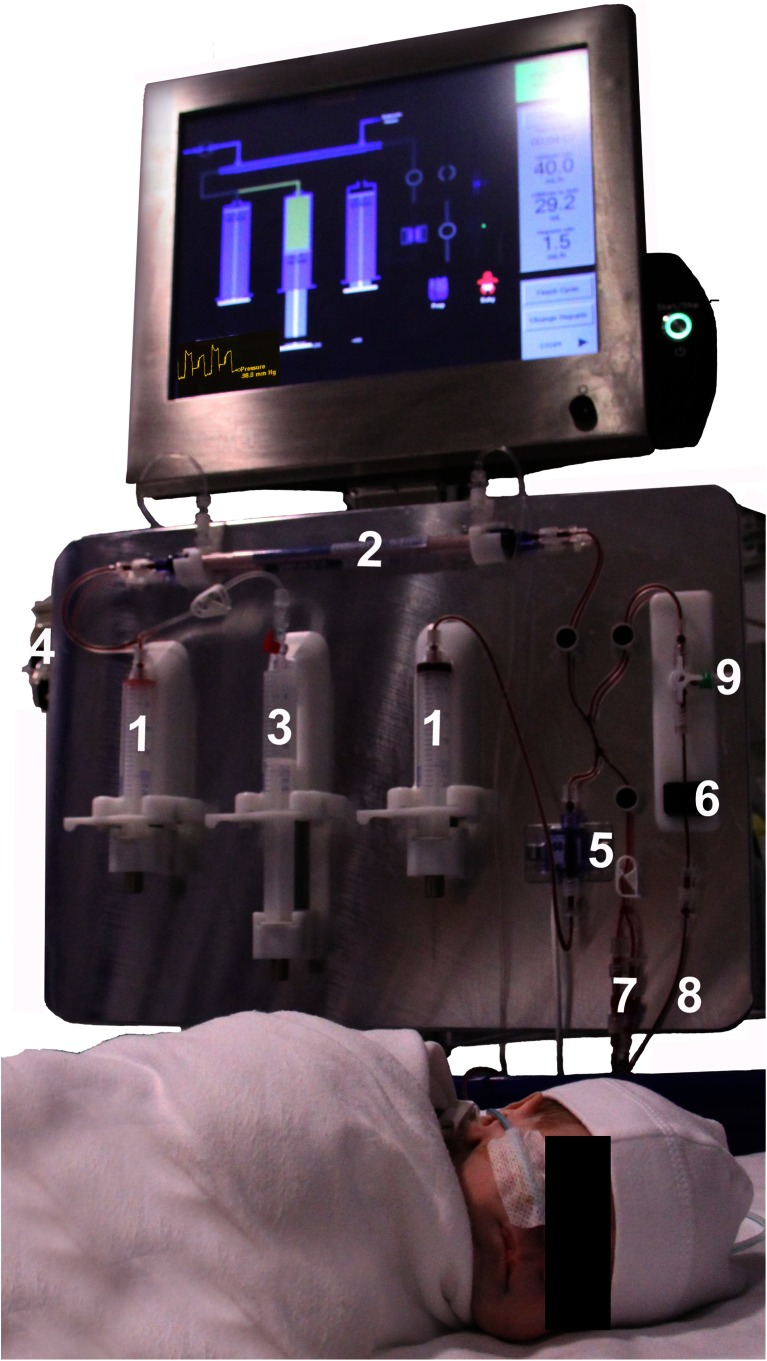
The Newcastle infant dialysis and ultrafiltration system (Nidus machine) in use. The numbered parts are described in the text. The parents gave their informed consent to use the photograph

This syringe-driven machine repeatedly withdraws 5 to 12.5 ml aliquots of blood from a single-lumen central venous line, passes and returns it across a dialysis filter, and then back to the baby. At a blood flow rate of 20 ml/min, this processes 5 ml of blood each minute. The circuit (Fig. 1) has two operating syringes (*1*), a high-flux polysulfone 0.045 m^2^ filter (*2*), a heparin syringe (*3*), pumped dialysate (*4*), a pressure transducer (*5*) and an air-detector (*6*), and self-primes with 4.3 ml of heparinised saline, giving a minimum operating volume of 9.3 ml. Ultrafiltration from 0 to 60 ml/h is precisely controlled in 3.2 μl steps by differential syringe movements. The circuit may be connected to a heparinised saline “dummy” [7], or the infant line [8], and blood may be sampled [9] for testing. The pressure-control pattern and exact circuit position are displayed on a touch-screen, which also informs the operator about warning and stop-alarm states. If the line is poor, the Nidus instantly slows its rate of blood withdrawal from the baby and informs the operator. It records its precise operating status and syringe positions every one-tenth of a second, and has battery back-up. The circuits have been designed for up to 24 h of continuous use.

#### Peritoneal dialysis

We performed manual PD using Tenckhoff catheters and bicarbonate-based dialysate, typically with 30-min cycles. In the piglets we used 40-ml/kg fill volumes, but to minimise the risk of acute hydrothorax in the babies we commenced at 10 ml/kg and aimed to increase to 40 ml/kg over 6 days if tolerated [19].

### Assessments

#### Chemical clearance measurements

We measured “instantaneous” dialysis clearances of urea, creatinine and phosphate using both treatment modalities by assaying their concentrations simultaneously in blood and in timed and measured effluent dialysate fluid samples, and using standard clearance formulae. We used enzymatic creatinine assays to avoid the effect of non-creatinine chromogens, such as glucose [20].

#### Ultrafiltration measurements—direct

For the Nidus, we determined the UF precision in vitro and in piglets by weighing the fluid removed to the nearest 0.1 g during isolated UF, and during dialysis and ultrafiltration by monitoring the combined weight of the fresh and waste dialysate bags. For PD, we measured the UF volume after every PD cycle. In both cases, we assessed UF adequacy by comparing the volumes of fluid removed during treatment with the prospectively agreed clinical targets.

#### Ultrafiltration measurements—indirect

In two clinically stable babies who underwent regular outpatient dialysis sessions of approximately 4-h on the Nidus, and subsequently on a conventional paediatric HD machine (Gambro AK200), we repeatedly estimated their fluid balance by measuring the weight changes to within 10 g at the start and finish of each session.

#### Statistical analysis

We expressed clearances as absolute values (ml/min), and per 1.73 m^2^ of body surface area, estimated from weight alone using Boyd’s self-adjusting power equation [21] (although this is likely to be an overestimate in piglets). We used paired *t* tests (P-t) to compare clearances within the same piglets, and independent *t* tests (I-t) between different babies or piglets. We compared UF values within the same piglets using the Wilcoxon matched pairs sign rank test (WMT), because their values were non-normal, and linear regression to compare the relation between clearance values and body weight. All tests were two-sided with probability (*p*) values expressed to one significant figure.

## Results

### Subjects studied

#### Piglets

Seven piglets (1.1 to 7.2 kg) were “treated” with both the Nidus and PD. Five were studied for 6 h each, and 2 were dialysed for 24 h.

#### Babies

Eleven babies were dialysed (6 boys; Table 1) at a median weight of 3.5 kg, and range 1.8 to 7.0 kg. An RCT was attempted, but only 2 babies were enrolled through this route, in part because of the lack of equipoise in this clinical setting. Of these 2 infants, 1 (case 10) only required PD for <24 h, and 1 (case 3) was only treated with PD for 10 h as it was not tolerated clinically. Nine infants were treated by the Nidus on compassionate grounds, 3 of whom also had periods of PD. Thus, 1 baby had PD alone, 6 had Nidus treatment alone, and 4 had both modalities, over a total of 192 h of PD, and 2,475 h of HD during 354 treatments.

**Table 1:** Clinical details of the 11 infants treated with dialysis

Case	Sex	Age (days)	Weight (kg)	Cause of renal failure	Prior ECMO	Reason for using haemodialysis and/ or PD	Haemodialysis		PD hourly dialysate flow
							Sessions	Hours	(ml/kg)
1	Male	3	1.8	ESRF, solitary MCD		Had colostomy for anal atresia	163	847	21
2	Female	58	3.3	ESRF, bilateral MCD		Fungal peritonitis with initial PD	11	66	28
3	Male	6	3.5	Post-cardiac surgery		RCT, but could not tolerate PD	5	75	20
4	Female	37	4.0	SVT causing ESRF	+	Had colostomy for NEC	158	1,236	
5	Female	6	4.1	Meconium aspiration, PPHN	+	Had NEC with abdominal distension	5	85	
6	Female	20	2.6	TBM and post-cardiac surgery	+	Open chest and high IPPV pressures	5	110	
7	Female	6	2.4	Methyl-malonic acidaemia		PD inefficient for ammonia removal	4	12	
8	Male	349	7.0	Post-cardiac surgery	+	Gut perforation with previous PD	1	24	
9	Male	5	3.1	Complex heart disease		Gut resection for NEC	1	12	
10	Male	27	4.0	Post-cardiac surgery	+	Started on PD and recovered promptly	0	0	20
11	Male	228	5.2	ESRF, renal dysplasia		Temporary PD failure	1	8	41
Totals							354	2,475	

*ECMO* extra-corporeal membrane oxygenation, *PD* peritoneal dialysis, *ESRF* end-stage (permanent) kidney failure, *MCD* multicystic dysplastic kidney, *RCT* randomised controlled dialysis trial, *SVT* supra-ventricular tachycardia, *PPHN* persistent pulmonary hypertension, *NEC* necrotising enterocolitis, *TBM* tracheo-broncho-malacia requiring stenting, *IPPV* intermittent positive pressure ventilation

### Chemical clearances

#### Piglets

**Fig. 2:**
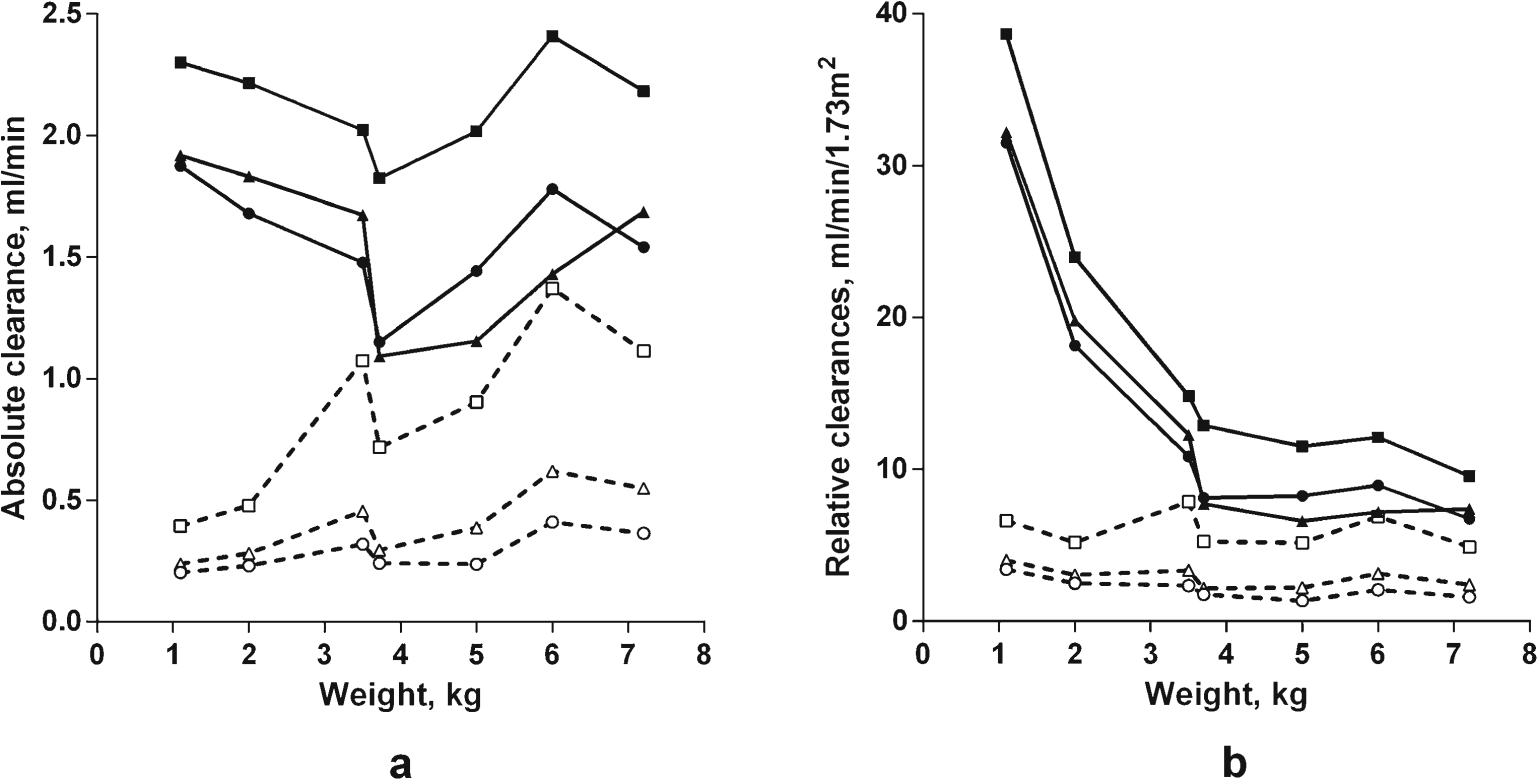
Chemical clearances in 7 piglets treated using the Newcastle infant dialysis and ultrafiltration system (Nidus, *solid symbols and lines*) and by peritoneal dialysis (PD, *open symbols and broken lines*), expressed **a** as absolute values, ml/min/piglet, and **b** relative to body surface area, ml/min/1.73 m^2^. In this case, the ultrafiltration was set at 40 ml/h for Nidus, and 3.86 % glucose dialysate was used for PD. *Squares* urea, *triangles* creatinine, *circles* phosphate

**Fig. 3:**
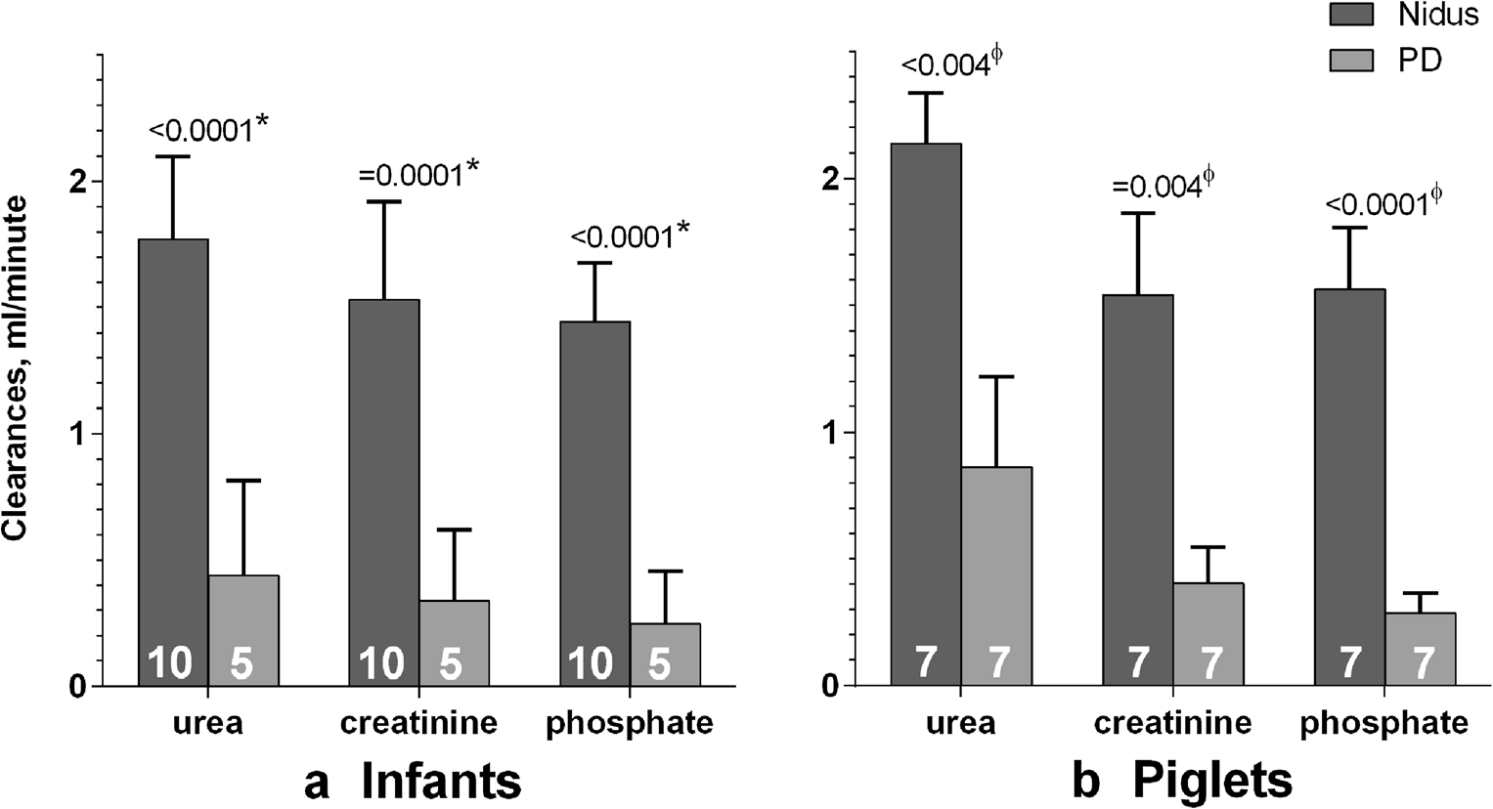
Absolute clearances of urea, creatinine and phosphate **a** in infants, and **b** in piglets, comparing those generated by the Nidus machine (*dark grey columns*) and by Peritoneal dialysis (*light grey*). For the babies, the values were the first ones measured in that child. The number of cases is shown on each column.*Comparing babies with unpaired *t* tests; ^ϕ^comparing each piglet using paired *t* tests

**Fig. 4:**
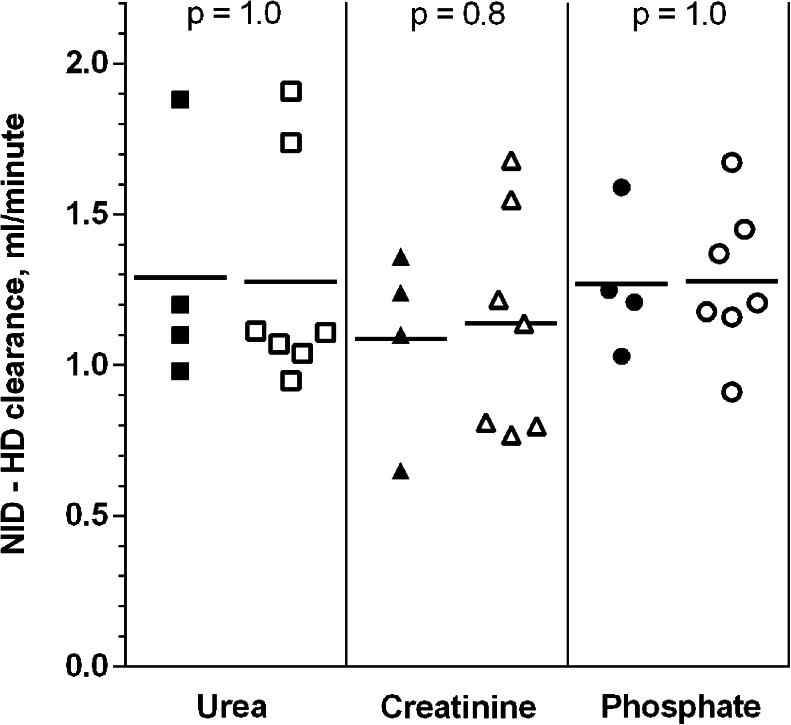
Differences between the absolute clearances of urea, creatinine and phosphate delivered by the Nidus machine and Peritoneal dialysis, ml/min, for the 4 babies (*solid symbols*) and 7 piglets (*open symbols*) that received both treatments. Means and individual values are shown. *p* values are for independent *t* tests comparing the baby and piglet clearances

The absolute clearances of urea, creatinine and phosphate delivered by the Nidus were similar in all the animals regardless of size (linear regressions vs weight, *p* = 0.3 to 0.9), and were considerably higher than the clearances delivered by PD (Fig. 2a; *p* ≤ 0.0002 for each, P-t). The absolute PD clearances for each chemical increased with body weight (*p* ≤ 0.04 for each, I-t), as would be expected when dialysis cycle volumes are prescribed per kilogram. The Nidus urea clearances (2.14 ml/min) were higher than for creatinine or phosphate (1.54 and 1.56 ml/min, *p* < 0.001 for each, P-t), consistent with polysulfone, having a small molecule sieving coefficient of 1, and urea also being distributed inside the blood cells. For PD, the clearance of urea was also higher than that of creatinine for the same reason (0.86 vs 0.40 ml/min, *p* = 0.005, P-t), but that of phosphate was lower (0.29 ml/min, *p* = 0.001, P-t), consistent with its slow movement across the peritoneum in older children [22]. The PD clearances per 1.73 m^2^ were the same for all sized piglets (linear regressions, *p* = 0.08 to 0.6), whereas the Nidus clearances were higher in the smaller animals (Fig. 2b). The absolute clearances of each chemical using the Nidus and PD are compared in Fig. 3b, and clearance differences for each animal are shown as open circles in Fig. 4.

#### Babies

**Fig. 5:**
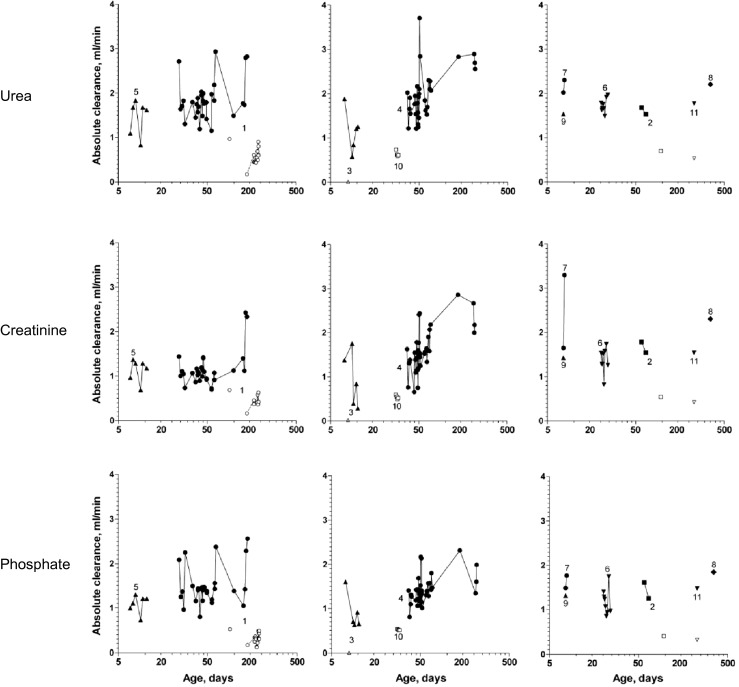
All of the absolute urea, creatinine and phosphate clearances measured in the babies, identified by their case number in Table 1. Treatments with the Nidus machine are shown with *solid symbols*, and Peritoneal dialysis treatments with *open symbols*. The same logarithmic time scale was used for all to improve the visual separation of individual babies’ values

The infants were dialysed for different periods of time according to their clinical needs, ranging from just one treatment session to many over periods of months (see Table 1 and Fig. 5, which displays all of our clearance measurements). To make comparisons with the piglet data, we only analysed the first measurements made for each baby. For clinical reasons, the PD cycle volumes and frequencies varied for these infants, which influenced chemical clearances, and the hourly per-kg throughput of dialysate are shown in Table 1.

Figure 3 shows that the Nidus delivered higher chemical clearances than PD (*p* ≤ 0.0001, I-t). The gains for each of the three chemical clearances from using HD rather than PD were also similar for the 4 infants who experienced both treatments, and close to the mean gains seen in the piglets (Fig. 4, *p* values 0.8 to 1.0, I-t).

### Ultrafiltration

#### Nidus

**Fig. 6:**
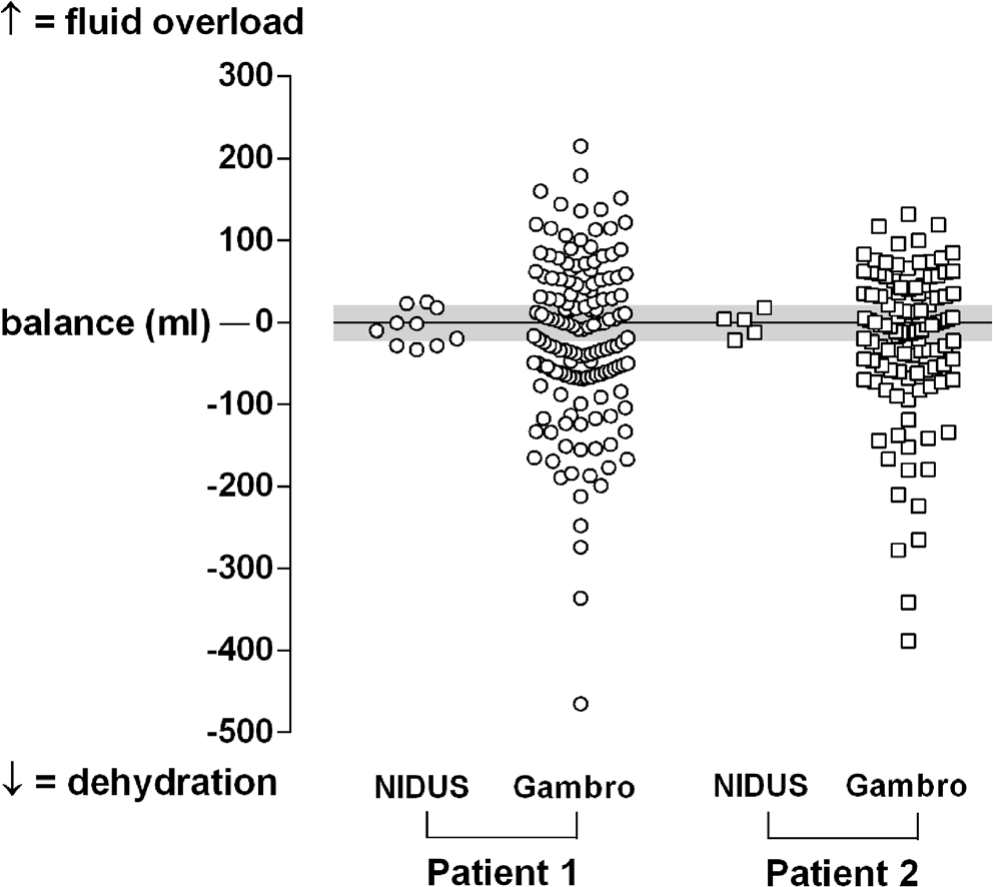
The estimated fluid balance errors in 2 babies who weighed about 6 kg, during routine outpatient dialysis and ultrafiltration sessions of 3 to 5 h, using the Nidus machine first, and then after changing them to a conventional paediatric haemodialysis machine (Gambro AK200). Fluid overload appears above the 0-line, dehydration below. The *grey line* indicates the expected error range of ±20 g from weighing the babies twice to the nearest 10 g

The UF was precise when measured directly by weighing the fluid removed with a detection limit of 0.25 %. Our repeated indirect UF assessments in 2 babies weighing about 6 kg showed good precision; the difference between their predicted and actual weights at the end of treatment had a standard deviation (SD) of ±17 g, and range of −33 to +25 g (Fig. 6). Using a Gambro AK200 paediatric HD machine, the variation was much greater, with an SD of ±96 ml (*p* = 0.003 for each child), and a range of −465 to +215 ml; saline boluses were required on several of these occasions for hypovolaemic symptoms.

#### Peritoneal dialysis

**Fig. 7:**
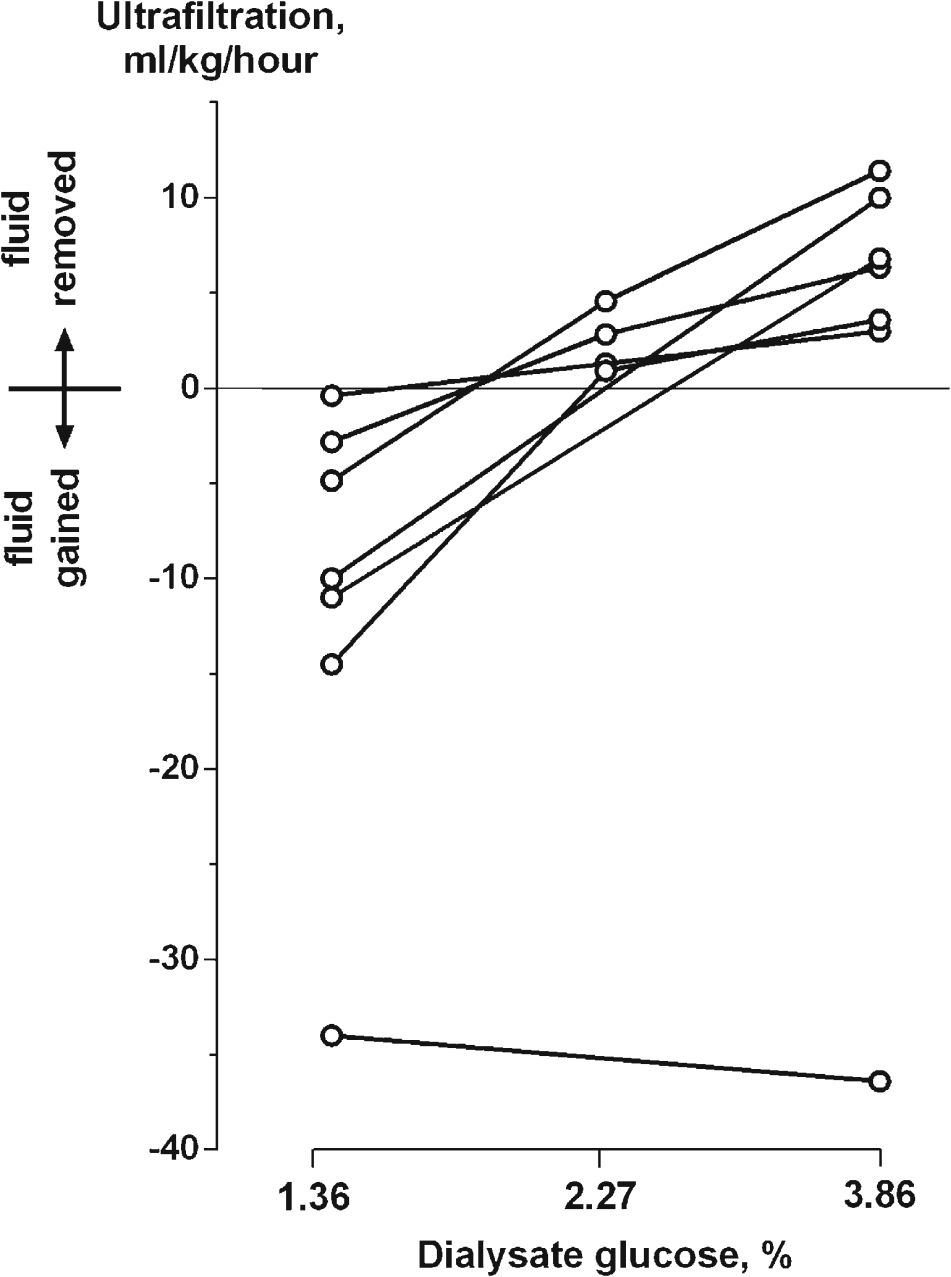
The ultrafiltration rates achieved in 7 piglets on peritoneal dialysis, using solutions with glucose concentrations of 1.36, 2.27 and 3.86 %. Positive values indicate fluid removal; negative ones indicate fluid gain

The piglets absorbed 0.4 to 14.5 ml/kg/h of water when dialysed with 1.36 % glucose dialysate, but 6 of the 7 lost from 3.0 to 11.4 ml/kg/h with 3.86 % glucose bags (Fig. 7). One animal absorbed very large fluid volumes with any strength bag. Failure to produce an adequate UF volume was a clinical problem in 4 of the 6 babies we treated with PD. When baby 1 had PD introduced (after closure of his colostomy), he continued to need the Nidus for fluid removal. Baby 2 lost UF control on PD after developing fungal peritonitis. Baby 11 was on long-term PD, but transiently lost his UF capacity when he developed large hydrocoeles, and he required Nidus treatment to prepare him for his herniotomy procedures. Baby 3 did not produce any ultrafiltrate or adequate dialysis during his 10 h on PD, during which time his peripheral perfusion and oxygen saturation fell and he became more unstable and difficult to ventilate. For this reason, the clinician ceased using PD. He improved after being restarted on treatment with the Nidus.

### Access, anticoagulation, haemolysis and air detection using the Nidus

Most babies treated acutely had 20-gauge, 10-cm venous cannulas that typically allowed blood withdrawal at 20 ml/min, although sometimes the Nidus needed to slow automatically with these lines. We used 6.5-Fr venous lines of 1.6 mm in internal diameter and with multiple side holes (Tessio) for children on long-term renal replacement. The performance of the circuits and dialysis filters remained unchanged during 36 h of continuous use.

We were able to successfully anticoagulate the circuit with heparin to an ACT to 120–160 s, whereas the ACT range typically used during HD is 180–220 s. There was no excessive bleeding. Early in the study, some circuits clotted if inappropriately high UF rates were set for the baby’s physiological state; see the section Nidus development below.

The plasma haemoglobin concentrations remained normal in all the piglets and babies, excluding significant haemolysis. There were two reported clinical incidents in baby 4 involving small air leaks into the circuit, both of which stopped the machine virtually instantaneously, isolating the baby’s blood line from risk, and triggered appropriate visual and audible alarms.

### Feedback and usability

The nursing and medical staff found the Nidus easy to operate and most preferred it to PD because of improved UF control and because it did not destabilise babies on commencement of therapy. Some parents commented on the machine’s simplicity and found that the onscreen display helped their understanding of the procedure, which they found reassuring. We downloaded the complete Nidus activity record after each patient’s treatment for teaching, machine development, and for clinical review by the medical, nursing and engineering teams.

### Nidus development

Before 2010, the early pilot machines had only been used by a small specialist medical and nursing team for babies under 4.5 kg, for continuous therapy on compassionate grounds when there was no alternative. We made new developments during this study in response to challenges that arose because the Nidus was also used on larger babies, sometimes for intermittent outpatient HD, and because of feedback we obtained when it was operated by a wider range of paediatric staff.

Early in the study, some filters clotted when attempts were made to remove fluid from oedematous babies who in retrospect were recognised to have had intravascular hypovolaemia. Initially, the device reported the rising filter operating pressure, which could be serially overridden, without directing the nurse to recognise its cause, or to reduce the UF rate setting. We solved this problem by introducing a software algorithm to automatically regulate the UF delivery rate according to changes in the dialyser’s operating pressure, and to indicate to the operator when this was happening.

In response to the clearance needs of babies up to 8 kg on intermittent dialysis, and to increase the efficiency of ammonia removal from babies with metabolic diseases, we re-engineered the Nidus during the latter part of the study so that it could be made to run both at its standard blood sampling rate of 20 ml/min, and at 45 ml/min. This corresponds approximately to the blood flow that can be obtained through a 6.5-Fr Tessio line, or a standard 16-gauge intravenous cannula (length 45 mm) at −300 mm Hg. This increased the chemical clearances proportionately (nearly 2½-fold) in an adult volunteer and in baby 4, in whom it was used for several dialysis sessions. We have subsequently increased the higher rate to 50 ml/min, and are currently modifying the filter geometry to optimise the operating pressures at this speed.

### Patient outcomes

#### Acute renal failure

Four of the 6 babies treated for acute renal failure with the Nidus recovered normal kidney function. Two had treatment withdrawn, one because she had evidence of brain damage owing to her pre-dialysis ammonia levels caused by methyl-malonic acidaemia, and one because he had an inoperable cardiac abnormality.

#### Chronic renal failure

One baby on chronic PD developed large hydrocoeles and failure of ultrafiltration. He was treated with the Nidus peri-operatively and then resumed PD successfully. Three other babies with permanent renal failure were transferred successfully to conventional HD on Gambro AK200 machines as they grew. In each case, growth faltering occurred as they reached about 5 kg. In the last of these (case 4) we responded to the stalled weight gain by increasing the dialysis prescription, both by increasing her treatment time, and by re-engineering the Nidus to operate at 45 ml/min. Her weight gain then accelerated, which allowed us to transfer her onto a conventional HD machine at 5.9 kg, and thus for her to be managed by her local paediatric dialysis centre. Case 1 developed liver cancer 2 years later and died. Case 2 has had a successful live donor transplant, and case 4 remains well on HD.

## Discussion

At the time of the study there were no machines licensed to deliver renal replacement for babies weighing between 800 g and 8 kg because of the imprecisions and circuit volume limitations that are inherent in conventional system designs. We have developed a unique device, the Nidus, which can provide haemodialysis and precise ultrafiltration across this weight range. The single-lumen access it requires is easier to achieve, it automatically slows its sampling rate from the baby if the access line is narrow and the blood flow rate of the baby’s line is uncoupled from the flow rate that the dialysis filter requires. Furthermore, it requires less anticoagulation than other extracorporeal circuits in clinical use. Because the circuit volume is less than 10 ml, it does not require blood priming, even for babies of 800 g, and its ultrafiltration control is delivered to microlitre precision.

Because the principles of delivering PD are technically simple [6, 10, 11], and it is relatively cheap to provide, it will continue to play a role in treating some acutely ill babies. However, the unpredictability of its fluid control, lower chemical clearances, and access complications may make the Nidus a better choice in some cases. Also, the Nidus can provide treatment for babies with necrotising enterocolitis, abdominal wall defects, or after abdominal surgery, when there are few other options. PD is likely to remain the treatment of choice for managing infants with chronic renal failure [8, 9], but when this is contraindicated or fails, the Nidus could provide a short- or long-term treatment bridge.

Commercially available HD and continuous veno-venous haemofiltration (CVVH) devices require dual-line or double-lumen access with relatively large central venous lines, and they demand a minimal supply of blood from the baby to prevent the filter from clotting. They also require relatively large extracorporeal circuits to incorporate pump inserts and a compliance chamber with a blood–air interface, which necessitates either blood priming for small babies, or priming with saline, which will induce a sudden haemodilution at connection, often followed by a blood transfusion to correct the induced anaemia. For the Gambro Prismaflex, a circuit volume of 60 ml [13] makes it desirable to blood prime the device for babies of <5 kg. The proposed USA miniaturised prototype will have a circuit volume of 43 ml,[23] and the CARPEDIEM circuit at 27 ml does not need blood priming for babies of >2.5 kg [16], but this machine has only just become available.

Ultrafiltration rates in conventional HD and CVVH systems are regulated by making adjustments to the pressure gradients operating across the dialysis membranes according to the fluid shifts that the machine assesses by continuously weighing the fresh and waste dialysate bags. The imprecision in this approximates ±30 ml per hour initially, falling to ±300 ml per day. These represent large errors in small babies, who frequently appear to be haemodynamically unstable on treatment, and often require saline boluses to treat episodes of clinical hypovolaemia.

## Conclusion

The unique design of the Nidus machine enables it to clear the blood of chemicals and fluid in babies weighing between 800 g and 8 kg, just using a single-lumen line, without blood priming, and with microlitre UF control. It may become the treatment modality of choice for many small babies with acute renal failure, and may have roles to play in managing infants with chronic renal failure. We anticipate that it will be commercially available within a few months. So far it has only been used in Newcastle, but we plan to undertake a prospective study of its use across the UK to further determine its contribution to managing infants requiring renal replacement therapies.
